# Interaction of Oral and Toothbrush Microbiota Affects Oral Cavity Health

**DOI:** 10.3389/fcimb.2020.00017

**Published:** 2020-02-04

**Authors:** Qingyao Shang, Yuan Gao, Ting Qin, Shuai Wang, Yan Shi, Tingtao Chen

**Affiliations:** ^1^The Key Laboratory of Oral Biomedicine, Department of Conservative Dentistry and Endodontics, The Affiliated Stomatological Hospital of Nanchang University, Nanchang, China; ^2^School of Stomatology, Nanchang University, Nanchang, China; ^3^National Engineering Research Centre for Bioengineering Drugs and the Technologies, Institute of Translational Medicine, Nanchang University, Nanchang, China

**Keywords:** brushing teeth, toothbrush, high-throughput sequencing, pathogens, oral microbiota

## Abstract

Tooth brushing is necessary to maintain oral health. Little research has been carried out to explore microbial diversity in toothbrushes and to study the potential impact of these bacteria on human health. In the present study, 20 participants were enrolled, and the microbial diversity in their oral cavity and toothbrushes was investigated using high-throughput sequencing. Our results indicate that 1,136 and 976 operational taxonomic units (OTUs) were obtained from groups CB (samples from toothbrushes of participants using traditional Chinese medicinal toothpaste) and AB (samples from toothbrushes of those using antibacterial toothpaste), respectively. The pathogens *Acinetobacter baumannii, Staphylococcus aureus*, and *Candida albicans* were identified on toothbrushes. The presence of these pathogens increases the chance for the host to get infectious diseases, neurodegenerative diseases, cardiovascular diseases, and cancers. Moreover, our *in vitro* results indicate that traditional Chinese medicinal toothpaste and antibacterial toothpaste can not only inhibit the growth of pathogens but also markedly inhibit the growth of probiotics *Lactobacillus salivarius* and *Streptococcus salivarius*. Therefore, the inhibitory effect of toothpaste on probiotics, together with the existence of pathogens in toothbrushes, indicates a potential risk of tooth brushing for people in a sub-healthy state.

## Introduction

Host to the second largest human microbial library, the oral cavity is composed of teeth, gingival sulcus, tongue, tonsils, and soft and hard palates, and provides a suitable growth environment for a large number of microorganisms (up to 700 species) (Xie et al., 2010). The oral microbiota is mainly composed of Firmicutes, Actinobacteria, Bacteroidetes, Fusobacteria, and Proteobacteria, which are indispensable for human health due to their ability to inhibit the proliferation of exogenous microorganisms and promote host homeostasis and defense (Bo et al., 2012).

Factors such as smoking, antibiotic abuse, mouthwash, and toothpaste can cause an imbalance in the oral microflora and eventually result in dental caries, gingivitis, periodontitis, candidiasis, endodontic infections, orthodontic infections, and oral cancer (Lin et al., 2008; Chen and Jiang, 2014). Dental caries and periodontal disease are common oral diseases which are caused by the dominance of acidogenic and acid-tolerating species (e.g., *Streptococcus mutans*) or the inflammation produced by these cariogenic bacteria (Aas et al., 2005; Lin et al., 2008). Worse, these oral diseases can lead to other systemic diseases. Pritchard et al. (2017) found that periodontal disease is an important causes of Alzheimer's disease (AD) due to epithelial cell barrier disruption and chronic infections. Whitmore and Lamont (2014) found that oral squamous cell carcinoma (OSCC) is closely related to an imbalance of oral bacteria.

Since the nineteenth century, toothbrushes have been widely used to suppress the growth of harmful bacteria, remove food residue, keep the breath fresh, protect enamel, and prevent and alleviate gingival inflammation (Fischman, 1997). However, the residual moisture and food debris residing in toothbrushes provide a suitable environment for pathogen growth, rendering them a potential risk for various oral diseases (Hays, 2006; Bik et al., 2010).

In the present study, a high-throughput sequencing method was applied to compare microbial diversity in the oral cavity and toothbrushes (used with traditional Chinese medicinal toothpaste and antibacterial toothpaste), to explore the potential risk of pathogens in toothbrushes and provide useful suggestions on oral health.

## Materials and Methods

### Ethical Statement

The study was approved by the Ethical Committee of the Stomatological Hospital of Nanchang University, and all participants provided written informed consent. All experiments were conducted in accordance with the approved guidelines.

### Selection of Participants

Participants aged between 18 and 30 were enrolled from the Medical College of Nanchang University. Their physical conditions, including age, weight, medical history, recent antibiotic use, smoking history, alcohol drinking, and oral health situation, were investigated by questionnaire survey. Twenty participants (10 males, 10 females), who were in good health, had not used antibiotics for a year, had no smoking history, did not drink alcohol, and practiced standard oral hygiene without the use of mouthwash, aged 20–27 (average 22 years old), were ultimately enrolled (Table 1).

**Table 1:** Information of participants enrolled in the present study including gender, age, and body weight.

	**Total**	**C**	**A**	***p*-value**
Participants (n)	20	10	10	—
Gender [n (%)]	10 (50)	5 (50)	5 (50)	1
Age (years)	22.65 (21.69–23.61)	22.00 (21.00–24.25)	21.50 (21.00–24.50)	0.74
Body weight (kg)	64.85 ± 10.36	65.57 ± 11.83	64.13 ± 9.26	0.77

*In total, 20 participants, who were in good health, had not used antibiotics for a year, had no smoking history, and did not drink alcohol, aged 20–27 (average 22 years old), were ultimately enrolled. Participants in group C brushed their teeth using traditional Chinese medicinal toothpaste. Participants in group A brushed their teeth using antibacterial toothpaste. The p-value was calculated by an independent-samples T-test using SPSS 17.0 software. P < 0.05 showed homogeneity of variance*.

### Sample Collection

The most representative types of toothpaste in China—traditional Chinese medicinal toothpaste [main active components: *Chondrus crispus, Coptis chinensis, Sarcandra glabra* (does not contain fluoride); no. 20160005, Cao Shan Hu Oral Care Products Co., Ltd, Jiang Xi] and antibacterial toothpaste (Crest Seven Good Effects toothpaste; main active components: sodium fluoride, stannous chloride; no. 20160024, Procter & Gamble Co., Ltd. in Guangzhou)—were used (Pinducciu et al., 1996; Mojon, 2002). The 20 participants were randomly divided into two groups—group C [brushed teeth using traditional Chinese medicinal toothpaste twice a day for 15 days (after breakfast and before sleep)] and group A [brushed teeth using antibacterial toothpaste twice a day for 15 days (after breakfast and before sleep)]. Bacteria were collected from the oral cavity and toothbrushes. Oral bacteria were collected by gargling using 10 ml of sterile saline solution, and toothbrush microorganisms were collected by vibrating the toothbrush in 10 ml of sterile saline solution in a 50 ml sterile Eppendorf (EP) tube using a vortex. Samples were divided into groups CO (oral samples from participants in group C, *n* = 10), CB (toothbrush samples from participants in group C, *n* = 10), AO (oral samples from participants in group A, *n* = 10) and AB (toothbrush samples from participants in group A, *n* = 10).

### DNA Extraction and High-Throughput Sequencing

Samples in each group were collected for bacterial DNA extraction by a bead beating method (Okpalugo et al., 2009) using genomic DNA kits (Tiangen Biotech Co., Ltd., Beijing, China) according to the manufacturer's instructions. Then, the concentration and quality of extracted DNA were detected via a spectrophotometer at 230 nm (A 230) and 260 nm (A 260) (NanoDrop; Thermo Fisher Scientific, Inc., Waltham, MA, USA). Then, the V4 region of the 16S rDNA genes of each sample was amplified using 515F/806R primers (515F, 5′-GTGCCAGCMGCCGCGGTAA-3′; 806R, 5′-GGACTACVSGGGTATCTAAT-3′), and PCR products were sequenced with an Illumina MiSeq platform (GenBank accession number PRJNA553856; Fang et al., 2016).

### Bioinformatics and Multivariate Statistics

Fast Length Adjustment of Short reads (FLASH) software (v1.2.7, http://ccb.jhu.edu/software/FLASH/) (Magoc and Salzberg, 2011) was used to pair sequences according to the overlapping bases. The UCLUST sequence alignment tool (Edgar, 2010) of Quantitative Insights into Microbial Ecology (QIIME) software was used to merge the previously obtained sequences and for partitioning of operational taxonomic units (OTUs) with 97% sequence similarity. R software was used to calculate the number of OTUs shared by each group and the proportion of unique OTUs. QIIME software was used to obtain a composition and abundance distribution table for each sample at the five classification levels. Using R software, a partial least squares discrimination analysis (PLS-DA) discriminant model was constructed based on the species abundance and sample grouping data. The variable importance in projection (VIP) coefficient was calculated for each species.

### Bacterial Isolation From the Oral Cavity and Toothbrushes

Bacteria from the oral rinse solution and toothbrushes were cultivated using brain heart infusion (BHI) broth on solid plates (37°C, 12 h), and bacteria were isolated by comparing bacterial colony size, color, and bacterial morphology. In the end, all isolates were further identified via molecular biological identification (Wang et al., 2015).

### Antibacterial Effect of Toothpaste

Toothbrushes were mixed with sterile water at a ratio of 1:3 (vol:vol) in a 50 ml sterile EP tube, and the supernatant was transferred to a new 50 ml sterile EP tube for further use.

Then, the tested strains—*Enterobacter hormaechei, Streptococcus salivarius, Staphylococcus aureus, E. cloacae, Enterococcus faecalis, L. salivarius*, and *Candida albicans*—were co-incubated with traditional Chinese medicinal toothpaste solution or antibacterial toothpaste solution for 4 h at 37°C, and a viable cell counting method was used to count the number of bacteria (Table 2; Deng et al., 2015).

**Table 2:** Sequencing results of isolates from oral rinse solution.

**Strain no**.	**Closest relative**	**Aerobic condition**	**Source**	**Similarity (%)**	**GeneBank no**.
1	*E. hormaechei*	Facultative anaerobic	Oral	99	HQ238708.1
2	*S. salivarius*	Facultative anaerobic	Oral	99	MH447009.1
3	*S. aureus*	Aerobic	Toothbrush	99	LR134351.1
4	*E. cloacae*	Facultative anaerobic	Oral	100	KT441077.1
5	*E. faecalis*	Facultative anaerobic	Oral	99	KE351689.1
6	*L. salivarius*	Facultative anaerobic	Oral	99	FJ384627.1
7	*C. albicans*	Aerobic	Toothbrush	99	BD267550.1

*Seven strains were isolated and identified from oral rinse solution: E. hormaechei, S. salivarius, S. aureus, E. cloacae, E. faecalis, L. salivarius, and C. albicans. The sequences were blasted with the NCBI database*.

### Statistical Analysis

Statistical analysis was carried out using R software. PLS-DA analysis was carried out according to the species abundance matrix and sample grouped data, and a discriminant model was constructed. Differences among groups were analyzed based on the Kruskal–Wallis test using Statistical Product and Service Solutions 20.0 (SPSS 20.0) software. The statistical significance was set at *P* < 0.05 for significant difference.

## Results

### Alpha Diversity of Microbiota in the Oral Cavity and Toothbrushes

To compare the microbes in the oral cavity and toothbrushes, 16S rDNA amplicon sequencing analysis was used to sequence the V4 hypervariable region, and the sequencing data were filtered to obtain valid data. The effective tags of all samples were clustered, and sequences with more than 97% similarity were considered as one OTU. In total, 1,680,600 usable raw sequences and 4,283 OTUs were obtained from all the samples, and the average number of OTUs in each group was 1,070 (data not shown).

**Figure 1:**
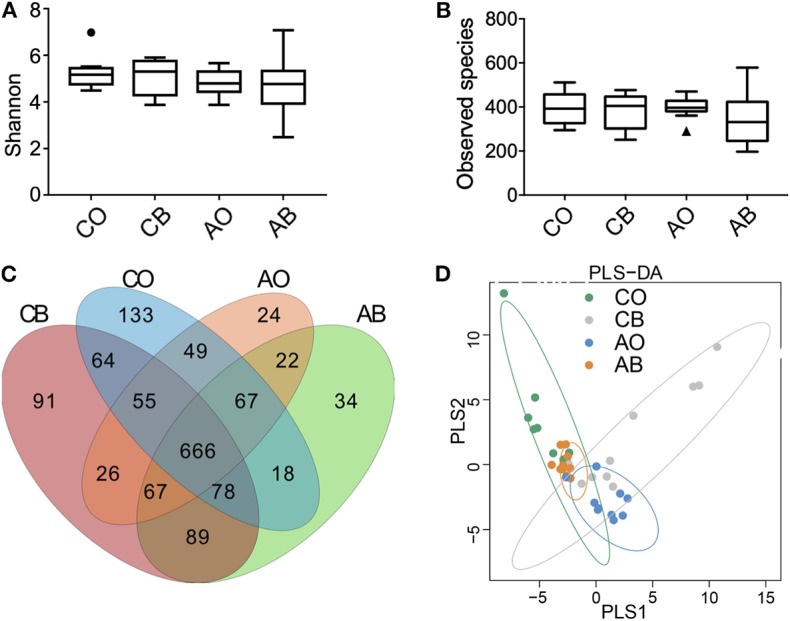
Indices of oral bacterial diversity. **(A)** Calculations of Shannon indices among groups CO, CB, AO, and AB; **(B)** calculations of species observed among groups CO, CB, AO, and AB; **(C)** scalar Venn diagrams among groups CO, CB, AO, and AB; **(D)** PLS-DA analysis of groups CO, CB, AO, and AB. CO, oral microbiota of people using traditional Chinese medicinal toothpaste (*n* = 10); CB, microbiota of toothbrushes corresponding to group CO (*n* = 10); AO, oral microbiota of people using antimicrobial toothpaste (*n* = 10); AB, microbiota of toothbrushes corresponding to group AO (*n* = 10).

As shown in Figure 1A, the Shannon index showed that the use of traditional Chinese medicinal toothpaste increased the microbial abundance in toothbrushes (CB) compared with the oral cavity (CO); microbial diversity in the oral cavity (AO) and toothbrushes (AB) using antibacterial toothpaste was almost the same. Figure 1B shows that use of traditional Chinese medicinal toothpaste also increased the number of bacterial species in toothbrushes (CB), while an obvious reduction was observed for toothbrushes (AB) compared with the oral cavity when antibacterial toothpaste was used (400 vs. 344).

The Venn diagram in Figure 1C indicates that 1,136, 976, 1,130, and 1,041 OTUs were obtained from groups CB, AB, CO, and AO, and the common OTUs (666) represented 58.63% (666/1,136) of CB, 68.24% (666/976) of AB, 58.94% (666/1,130) of CO, and 63.98% (666/1,041) of AO, respectively. In addition, the opportunistic pathogens *Burkholderia cepacia* and *Pseudomonas pseudoalcaligenes* were identified from groups CB and AB, and a large number of the opportunistic pathogens *Stenotrophomonas maltophilia* and *Acinetobacter baumannii* (reported as clinically drug-resistant) were identified from group AB (Moore et al., 1999; Gales et al., 2001).

In Figure 1D, we can see that the dots in groups CO and CB are far away from each other, while those in AO and AB are gathered together, indicating that the strong antimicrobial activity of antibacterial toothpaste had an impactful convergent effect on microbial diversity.

### Beta Diversity of Microbiota in Oral Cavity and Toothbrush

**Figure 2:**
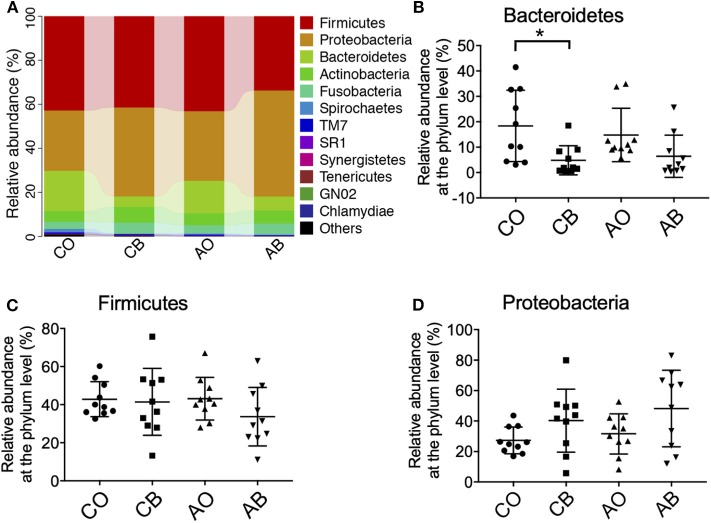
Ratios of the known bacteria in groups CO, CB, AO, and AB at the phylum level **(A)**. Relative abundance of Bacteroidetes **(B)**, Firmicutes **(C)**, and Proteobacteria **(D)** in groups CO, CB, AO, and AB. CO, oral microbiota of people using traditional Chinese medicinal toothpaste (*n* = 10); CB, microbiota of toothbrushes corresponding to group CO (*n* = 10); AO, oral microbiota of people using antimicrobial toothpaste (*n* = 10); AB, microbiota of toothbrushes corresponding to group AO (*n* = 10). Data are presented as means ± SD; *P < 0.05 by Kruskal–Wallis.

Based on the weighted UniFrac distance at phylum level, data for the top 12 microorganism populations were analyzed to check the similarity among different groups (24). As presented in Figure 2A, Firmicutes, Proteobacteria, and Bacteroidetes were the three dominant phyla and accounted for >85% of the total sequencing number in all groups; the percentage of Firmicutes, Proteobacteria, and Bacteroidetes was 42.84% vs. 41.46% vs. 43.14% vs. 33.70%, 27.34% vs. 40.33% vs. 31.61% vs. 48.17%, and 18.34% vs. 4.84% vs. 14.80% vs. 6.40% in groups CO, CB, AO, and AB, respectively. Use of traditional Chinese medicinal toothpaste reduced the abundance of Bacteroidetes (CB vs. CO = 4.84 vs. 18.34%) and increased the abundance of Proteobacteria (CB vs. CO = 40.33 vs. 27.34%; Figures 2B,D). Antibacterial toothpaste obviously reduced the abundance of Bacteroidetes (AB vs. AO = 6.40 vs. 14.80%) and Firmicutes (AB vs. AO = 33.70 vs. 43.13%), while it increased the abundance of Proteobacteria (AB vs. AO = 48.16 vs. 31.61%) compared with group AO (Figures 2B–D).

**Figure 3:**
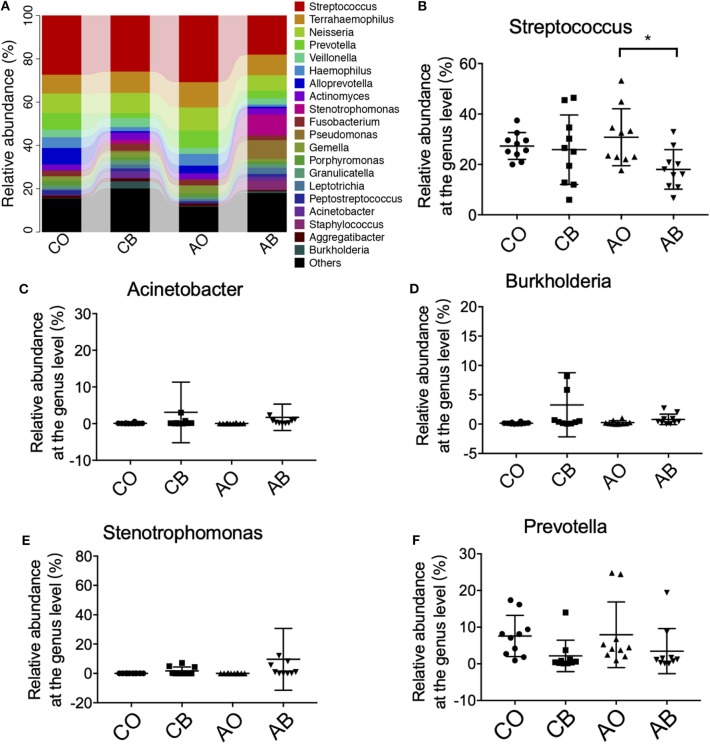
Ratios of the known bacteria in groups CO, CB, AO, and AB at the genus level. **(A)** Relative abundance of *Streptococcus* **(B)**, *Acinetobacter* **(C)**, *Burkholderia* **(D)**, *Stenotrophomonas* **(E)**, and *Prevotella* **(F)** in groups CO, CB, AO, and AB. CO, oral microbiota of people using traditional Chinese medicinal toothpaste (*n* = 10); CB, microbiota of toothbrushes corresponding to group CO (*n* = 10); AO, oral microbiota of people using antimicrobial toothpaste (*n* = 10); AB, microbiota of toothbrushes corresponding to group AO (*n* = 10). Data are presented as means ± SD; *P < 0.05 by Kruskal–Wallis.

At genus level, *Streptococcus, Terrahaemophilus, Neisseria*, and *Prevotella* were dominant in all groups (Figure 3A), and traditional Chinese medicinal toothpaste and antibacterial toothpaste hardly affected the abundance of *Neisseria, Actinomyces, Stenotrophomonas, Acinetobacter*, or *Burkholderia* in all groups (Figures 3B–F). However, it seems that use of antibacterial toothpaste in group AO could increase the number of *Streptococcus* in the oral cavity compared with traditional Chinese medicinal toothpaste (Figure 3).

### Potential Risk of Microbial Changes in the Oral Cavity and Toothbrushes for Human Diseases

**Figure 4:**
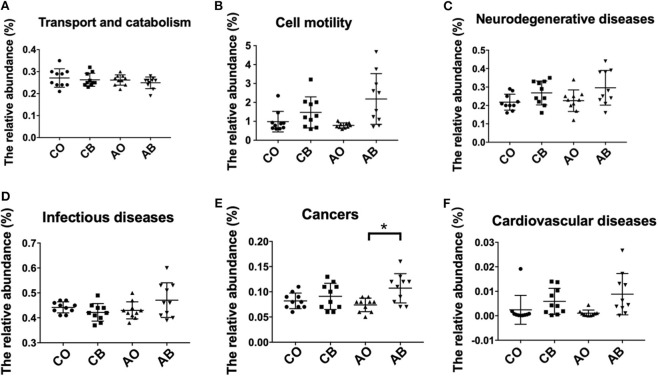
Relative abundance of groups CO, CB, AO, and AB using PICRUSt based on the KEGG database. Classification based on cellular process—transport and catabolism **(A)**, cell motility **(B)**. Classification based on human disease—neurodegenerative diseases **(C)**, infectious diseases **(D)**, cancers **(E)**, cardiovascular diseases **(F)**. CO, oral microbiota of people using traditional Chinese medicinal toothpaste (*n* = 10); CB, microbiota of toothbrushes corresponding to group CO (*n* = 10); AO, oral microbiota of people using antimicrobial toothpaste (*n* = 10); AB, microbiota of toothbrushes corresponding to group AO (*n* = 10). Data are presented as means ± SD; *P < 0.05 by Kruskal–Wallis.

We evaluated the potential risk of microbial diversity in the oral cavity and toothbrushes for human diseases based on the KEGG database. As shown in Figure 4, there were no significant differences in transport and catabolism capability among groups CO, CB, AO, and AB (Figure 4A), while bacteria in toothbrushes (from both traditional Chinese medicinal toothpaste and antibacterial toothpaste groups) greatly enhanced cell motility in the host (Figure 4B) and the chance of the host suffering from neurodegenerative diseases, infectious diseases, cancers, and cardiovascular diseases (Figures 4C–F). It seems that the high abundance of pathogenic bacteria in toothbrushes obviously increased the potential risk for human health.

### Bacterial Isolation and Antibacterial Effect of Toothpastes on Isolates

To further study the antibacterial effect of toothpastes on oral bacteria, the plate method and molecular biological identification were used to isolate bacteria from the oral cavity and toothbrushes. As shown in Table 1, *E. hormaechei, S. salivarius, S. aureus, E. cloacae, E. faecalis, L. salivarius*, and *C. albicans* were screened, of which *E. hormaechei, S. aureus, E. cloacae, E. faecalis*, and *C. albicans* are pathogenic bacteria, and *L. salivarius* and *S. salivarius* are beneficial bacteria.

**Figure 5:**
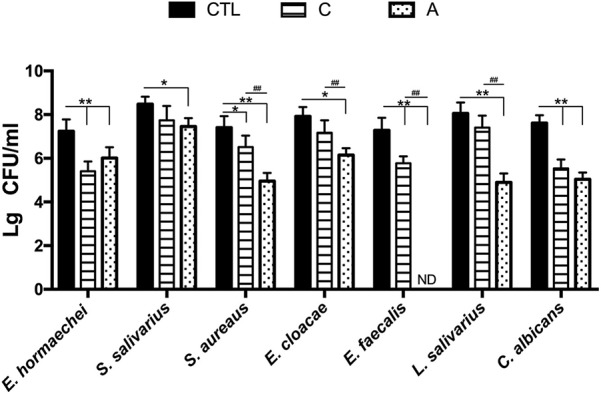
Inhibitory action of traditional Chinese medicinal toothpaste and antimicrobial toothpaste on isolated strains. CTL: PBS buffer; C, 20% concentration of traditional Chinese medicinal toothpaste; A, 20% concentration of antimicrobial toothpaste. CO, oral microbiota of people using traditional Chinese medicinal toothpaste (*n* = 10); CB, microbiota of toothbrushes corresponding to group CO (*n* = 10); AO, oral microbiota of people using antimicrobial toothpaste (*n* = 10); AB, microbiota of toothbrushes corresponding to group AO (*n* = 10). *P < 0.05, **P < 0.01, ^#^*P* < 0.05, ^##^*P* < 0.01. Data are presented as means ± SD; *P < 0.05 vs. control group (CTL group) by ANOVA, ^#^*P* < 0.05 vs. C group by Kruskal–Wallis.

When testing the antibacterial effect of toothpastes, we found that antibacterial toothpaste significantly inhibited the growth of *E. hormaechei* (A vs. CTL = Lg 6.01 vs. Lg 7.23, *p* < 0.01), *S. salivarius* (A vs. CTL = Lg 7.45 vs. Lg 8.48, *p* < 0.05), *S. aureus* (A vs. CTL = Lg 4.95 vs. Lg 7.40, *p* < 0.01), *E. cloacae* (A vs. CTL = Lg 6.14 vs. Lg 7.92, *p* < 0.01), *E. faecalis* (A vs. CTL = ND vs. Lg 7.28, *p* < 0.01), *L. salivarius* (A vs. CTL = Lg 4.89 vs. Lg 8.05, *p* < 0.01), and *C. albicans* (A vs. CTL = Lg 5.04 vs. Lg 7.61, *p* < 0.01). Although the antibacterial effect of traditional Chinese medicinal toothpaste was inferior, it also obviously inhibited the growth of *E. hormaechei* (C vs. CTL = Lg 5.40 vs. Lg 7.23, *p* < 0.01), *S. aureus* (C vs. CTL = Lg 6.51 vs. Lg 7.40, *p* < 0.05), *E. faecalis* (C vs. CTL = Lg 5.77 vs. Lg 7.28, *p* < 0.01), and *C. albicans* (C vs. CTL = Lg 5.53 vs. Lg 7.61, *p* < 0.01; Figure 5).

## Discussion

The long-term use of mouthwash, antibacterial toothpaste, and throat tablets can cause dysbacteriosis and further endanger oral health (Hays, 2006; Bik et al., 2010). Research has indicated that microorganisms in toothbrushes might have a potential risk to cause a variety of diseases (Hays, 2006), but little research has been carried out to explore the interaction of microbiota between the oral cavity and toothbrush.

In the present study, high-throughput sequencing was used to explore microbial diversity in the oral cavity and toothbrushes. The oral cavity and toothbrushes had a similar Shannon index and species observed, and the common OTUs (666) represented 58.63, 68.24, 58.94, and 63.98% of the population in groups CB, AB, CO, and AO. Therefore, the abundant nutrients and frequent microbial communication resulted in a high number and variety of species of bacteria in toothbrushes (Figure 1). Although some ingredients (e.g., honeysuckle, *Sarcandra glabra*, pseudo-ginseng, mint) contained in traditional Chinese medicinal toothpaste possess and anti-inflammatory effects, it seems these ingredients allow for a greater abundance of bacteria compared with antibacterial toothpaste containing antibacterial ingredients (e.g., sodium fluoride, stannous chloride; Figure 1D). In addition, some opportunistic pathogens, e.g., *A. baumannii* (which causes wound infection, sepsis, and even leads to death), *B. cepacia* (which causes cystic fibrosis in human lungs), and *S. maltophilia* (which causes iatrogenic infection and respiratory infections), were identified in groups AB and CB (Moore et al., 1999; Gales et al., 2001). This means these pathogens in toothbrushes may cause serious infectious diseases in low-immunity populations.

At phylum level, we found greater abundance of Proteobacteria in groups CB and AB. The phylum Proteobacteria contains various human pathogens, e.g., *Helicobacter, Neisseria, Brucella*, and *Rickettsia*, and they are reported to have a strong connection with metabolic disorders, inflammatory bowel disease, and lung disease (Wade, 2013; Chen and Jiang, 2014; Rizzatti et al., 2017; Vester-Andersen et al., 2017). At genus level, higher levels of *Actinomyces, Burkholderia*, and *Stenotrophomonas* were identified in groups CB and AB. *Acinetobacter* species are ubiquitous organisms widely distributed in nature as important opportunistic pathogens causing a range of nosocomial infections, including pneumonia, bacteraemia, secondary meningitis, urinary tract infections, and surgical wound infections (Gales et al., 2001). *Burkebacteria* were originally part of the *pseudogencin*, and *B. cepacia* and *B. pseudomallei* in this genus have harmful effects on human health because of their characteristics of resistance to antibiotics and high motility (Moore et al., 1999). *Stenotrophomonas* is famous for *S. maltophilia* which is an important nosocomial pathogen associated with infections of compromised individuals (Gales et al., 2001). *S. maltophilia* is associated with infections of the respiratory tract and causes bacteraemia, endocarditis, and urinary tract infections. It is intrinsically resistant to multiple antibiotics and disinfectants, and clinical isolates often display high-level multidrug resistance (Zhang et al., 2000). Interestingly, we also found a lower abundance of *Streptococcus* and *Prevotella* in toothbrushes than in the oral cavity. As we know, *Streptococcus* and *Prevotella* are dominant pathogens for inducing oral diseases; therefore, the residual ingredients in toothbrushes can better inhibit their growth because there are more antimicrobial ingredients in toothbrushes than in the oral cavity due to the high fluidity of the oral cavity. Furthermore, our bioinformatics prediction results indicate that the bacteria in toothbrushes greatly enhanced cell motility, neurodegenerative diseases, cancers, and cardiovascular diseases when traditional Chinese medicinal toothpaste was used, and the antimicrobial toothpaste greatly enhanced the potential for cell motility, neurodegenerative diseases, infectious diseases, cancers, and cardiovascular diseases compared with the bacteria in the oral cavity (Figure 4). However, as limited data are provided by high-throughput sequencing, the shotgun metagenomic method is needed to further confirm these results.

To further discuss the potential effects of toothpastes on bacterial growth, the viable counting method was applied and *E. hormaechei, S. salivarius, S. aureus, E. cloacae, E. faecalis, L. salivarius*, and *C. albicans* were isolated from the oral cavities and toothbrushes. Our results indicate that traditional Chinese medicinal toothpaste and antimicrobial toothpaste possess sound inhibitory effects on all tested strains (Figure 5). *E. hormaechei* is a nosocomial pathogen that can infect vulnerable hospitalized patients and can be transmitted from patient to patient when infection control techniques are inadequate (Wenger et al., 1997). The traditional Chinese medicinal toothpaste had a better inhibitory effect on *E. hormaechei*. *S. aureus*, including methicillin-resistant *S. aureus* (MRSA), is an important food-borne pathogenic bacterium associated with significant morbidity and mortality. *C. albicans* is primarily a commensal organism in the oral cavity, digestive tract, and genital regions of healthy individuals and yet is responsible for mucosal candidiasis, such as thrush and angular keratitis, as well as visceral candidiasis, often deadly infections (Rai et al., 2019). We also tested two oral probiotics, *L. salivarius*, and *S. salivarius*. As a typical anti-allergic probiotic, *L. salivarius* helps reduce the production of specific IgE antibodies in serum, promotes the secretion of interferon (IFN-γ) in spleen cells, and enhances the TH1-type immune response to regulate the TH2-type immune response which is overactive due to allergies (Iwamoto et al., 2010; Li et al., 2010). *S. salivarius* can produce bacteriocin, which has an inhibitory effect on bad breath-related bacteria, especially anaerobic bacteria which decompose to produce sulfides, emitting a foul odor and producing bad breath (Volpe et al., 1969; Iwamoto et al., 2010). Studies have shown that people with bad breath have less *S. salivarius* in the mouth (Iwamoto et al., 2010). Although both traditional Chinese medicinal toothpaste and antibacterial toothpaste effectively reduced pathogenic bacteria, they also suppressed the oral probiotics which destroyed the stability of the oral microbiota and damaged oral health, especially the antibacterial toothpaste.

## Conclusion

**Figure 6:**
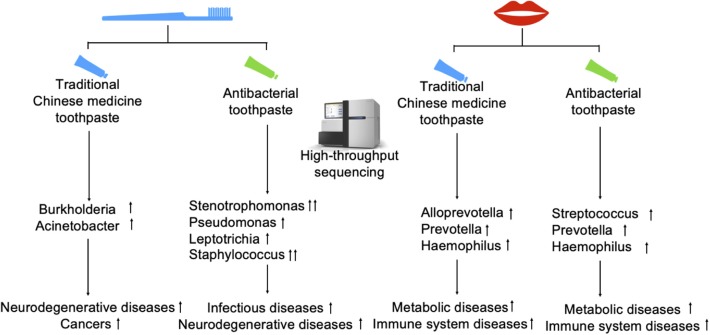
Flow chart of the present study. Toothbrush and oral samples were obtained from the participants treated with traditional Chinese medicinal toothpaste and antibacterial toothpaste, respectively. A high-throughput sequencing method was used to analyse species richness. Genera showing a significant increase compared with other groups are shown with ↑; those showing a highly significant increase are shown with ↑↑. The increased risks of human disease for each group are shown.

In the present study, we compared microbial diversity in the oral cavities and toothbrushes, and found a large number of bacteria in toothbrushes, including some pathogens. Pathogens existing in toothbrushes may transfer to the oral cavity and enhance the risk of neurodegenerative diseases, cancers, and cardiovascular diseases (Figure 6). Moreover, the strong antimicrobial effect of toothpaste on beneficial oral bacteria may also disturb the oral microbial system. Here, we compared the microbiota between oral cavities and toothbrushes, and explored the potential risk of bacteria in toothbrushes, which provides useful data on oral health. However, sampling by gargling carries a risk of contamination from the throat or upper aerodigestive ways; therefore, a more suitable sampling method for oral bacteria should be used (Santigli et al., 2017).

## Data Availability Statement

Publicly available datasets were analyzed in this study. This data can be found here: GenBank accession number PRJNA553856.

## Ethics Statement

The studies involving human participants were reviewed and approved by the Ethical Committee of the Stomatological Hospital of Nanchang University and all participants provided written informed consent. All experiences were conducted in accordance with the approved guidelines. The patients/participants provided their written informed consent to participate in this study.

## Author Contributions

TC designed the experiments. TC, YS, QS, YG, TQ, and SW analyzed the data and wrote the manuscript. QS, YG, TQ, and SW performed the experiments. All authors discussed the results and commented on the final manuscript.

### Conflict of Interest

The authors declare that the research was conducted in the absence of any commercial or financial relationships that could be construed as a potential conflict of interest.
